# Short-Term Clinical Outcomes and Systemic Inflammatory Biomarker Responses Following Platelet-Rich Plasma Injection in Knee Osteoarthritis

**DOI:** 10.3390/life16071097

**Published:** 2026-06-30

**Authors:** Viorela Mihaela Ciortea, Alina Deniza Ciubean, Titus Vari, Irina Motoașcă, Oana Valentina Harșa, Theodor Popa, Mădălina-Gabriela Iliescu, Liliana-Elena Stanciu, Florina-Ligia Popa, Tudor-Ștefan Ciortea, Liviuta Budișan, Cosmina Ioana Bondor, Laszlo Irsay

**Affiliations:** 1 Department of Physical Medicine, Balneotherapy and Rehabilitation, “Iuliu Hațieganu” University of Medicine and Pharmacy, 400347 Cluj-Napoca, Romania; viorela.ciortea@umfcluj.ro (V.M.C.); laszlo.irsay@umfcluj.ro (L.I.); 2 Department of Rehabilitation Medicine, Clinical Rehabilitation Hospital, 400347 Cluj-Napoca, Romania; oanaharsa@yahoo.com (O.V.H.);; 3 Doctoral School, Faculty of Medicine, “Iuliu Hațieganu” University of Medicine and Pharmacy, 400347 Cluj-Napoca, Romania; 4 Department of Rehabilitation Medicine, Faculty of Medicine, “Ovidius” University of Constanta, 900470 Constanta, Romania; madalina.iliescu@365.univ-ovidius.ro (M.-G.I.); lilianastanciu77@yahoo.com (L.-E.S.); 5 Department of Physical Medicine and Rehabilitation, Faculty of Medicine, “Lucian Blaga” University of Sibiu, 550245 Sibiu, Romania; 6 Faculty of Medicine, “Iuliu Hațieganu” University of Medicine and Pharmacy, 400347 Cluj-Napoca, Romania; 7 Genomics Department, MEDFUTURE Institute of Biomedical Research, “Iuliu Hatieganu” University of Medicine and Pharmacy, 23 Marinescu Street, 400337 Cluj-Napoca, Romania; liviuta.petrisor@umfcluj.ro; 8 Department of Medical Informatics and Biostatistics, Faculty of Medicine, “Iuliu Hatieganu” University of Medicine and Pharmacy, 400347 Cluj-Napoca, Romania

**Keywords:** knee osteoarthritis, platelet-rich plasma, inflammation, pain, mobility, quality of life

## Abstract

Background: Knee osteoarthritis (KOA) is a degenerative joint disorder characterized by chronic pain, functional limitation, and low-grade inflammation. While platelet-rich plasma (PRP) has gained traction as a biologic therapy, the relationship between short-term clinical outcomes and systemic inflammatory biomarker dynamics remains poorly understood in routine clinical settings. Methods: This prospective observational study evaluated 40 patients with grade 2–3 KOA receiving a single ultrasound-guided intra-articular PRP injection. Clinical outcomes via the Visual Analog Scale (VAS), the Western Ontario and McMaster Universities Osteoarthritis Index (WOMAC), and the Short Form-36 (SF-36), alongside serum inflammatory markers (IL-1β, IL-8, IL-18, TNF-α, erythrocyte sedimentation rate, and C-reactive protein), were assessed at baseline and 4 weeks post-injection using paired tests and multivariable regression. Results: At 4 weeks, clinical scores improved significantly (all *p* < 0.001). Circulating IL-8 and TNF-α levels decreased significantly (*p* = 0.009 and *p* = 0.014, respectively), whereas IL-1β and IL-18 variations were non-significant. Baseline cytokines did not predict clinical outcomes, but a significant association emerged between ΔIL-18 and ΔWOMAC (*p* = 0.032). Conclusions: A single intra-articular PRP injection was associated with short-term clinical improvements and selected reductions in circulating IL-8 and TNF-α, although the clinical significance of these biomarker changes remains uncertain. Given the observational design and short follow-up, these preliminary findings require confirmation in larger controlled trials.

## 1. Introduction

Knee osteoarthritis (KOA) is a leading chronic musculoskeletal disorder worldwide, driving chronic pain, functional limitation, and impaired quality of life [1]. Long viewed as a simple wear-and-tear condition, KOA is now recognized as a complex whole-joint disease [2,3]. In its early stages, mechanical overload and low-grade inflammation disrupt the extracellular matrix, leading to a loss of proteoglycans and a significant decrease in the mechanical strength of the cartilage [4]. While conventional conservative strategies—such as physical rehabilitation, NSAIDs, and corticosteroid injections—offer temporary symptomatic relief [5,6], they fail to halt this progressive structural decline and frequently carry systemic adverse risks [7,8].

Consequently, clinical interest has shifted toward orthobiologic approaches. Among these, platelet-rich plasma (PRP), an autologous blood-derived product, has emerged as a widely investigated therapy for mild-to-moderate KOA. PRP utilizes a concentrated reservoir of growth factors, cytokines, and chemokines stored within platelet α-granules [9,10,11]. Upon intra-articular injection, this bioactive milieu is thought to promote immunomodulation, recruit reparative cells, and support joint homeostasis, potentially slowing degenerative processes without the side effects typical of off-the-shelf pharmaceuticals [9].

While systematic reviews show that PRP provides superior, longer-lasting clinical results at 6 to 12 months compared to hyaluronic acid or corticosteroids [9,12], interpreting the broader literature remains difficult due to profound product heterogeneity [9]. Depending on the preparation method, blood derivatives exhibit vast variations in leukocyte content, centrifugation protocols, and final platelet concentrations [9]. The in vivo role of leukocytes remains highly debated regarding their pro-inflammatory potential [13,14], and recent evidence emphasizes that an absolute count of 10 billion platelets may be a critical threshold for therapeutic success [15]. This lack of standardization often limits the ability to draw definitive conclusions across trials [9].

This heterogeneity underscores a significant translational research gap. While controlled studies have demonstrated that PRP has been associated with lower synovial fluid levels of pro-inflammatory cytokines [15,16], real-world observational data tracking these physiological changes—particularly within the systemic circulation—remain sparse. Cytokines such as interleukin (IL)-1β, IL-8, IL-18, and tumor necrosis factor-alpha (TNF-α) are central to synovial inflammation [17,18,19] and pain sensitization [20,21]. Mechanistically, IL-1β and TNF-α act as principal catabolic drivers within the joint, significantly upregulating matrix metalloproteinases (MMPs) and aggrecanases that accelerate articular cartilage matrix degradation [17,18,19]. Concurrently, chemokines such as IL-8 augment the localized inflammatory microenvironment [22], while the cumulative elevation of these synovial signaling molecules directly or indirectly sensitizes primary afferent nociceptors, driving joint hypersensitivity and chronic pain intensification [20,21]. Although early clinical improvements are documented, the immediate, short-term systemic inflammatory biomarker responses following PRP administration remain poorly characterized.

Therefore, the aim of this prospective exploratory study was to evaluate short-term changes in pain, functional status, quality of life, and systemic inflammatory biomarkers following a single intra-articular PRP injection in patients with mild-to-moderate knee osteoarthritis in a routine clinical setting.

## 2. Materials and Methods

### 2.1. Patient Selection

This prospective, observational cohort study evaluated 40 patients at the Cluj-Napoca Clinical Rehabilitation Hospital, Department of Rehabilitation Medicine, between October and December 2025. Eligible participants were aged 47–77 years with a diagnosis of primary, unilateral or bilateral knee osteoarthritis (grade 2–3 according to the Kellgren–Lawrence classification) and a history of chronic knee pain exceeding 4 on a 0–10 Visual Analog Scale (VAS) for at least six months. For patients presenting with bilateral knee osteoarthritis, only a single target knee—specifically the one reported by the patient as having greater pain and more limited function —was selected for the study and received the injection.

Exclusion criteria comprised secondary knee osteoarthritis; a history of inflammatory rheumatological diseases, gout, or prior knee trauma or surgery; intra-articular injections within the preceding three months; active malignancy; hematological or immunological disorders; clinical or laboratory signs of acute infection; and coagulation disorders.

All participants provided written informed consent prior to enrollment. Baseline data collection included age, sex, height, weight, and relevant comorbidities. The structural severity of knee osteoarthritis was determined via standard anteroposterior and lateral knee radiographs obtained within three months prior to study entry.

### 2.2. Blood Sampling and Laboratory Analysis

On the morning of the procedure (T0), five fasting venous blood tubes were collected from each participant. Three tubes were allocated for PRP preparation (detailed below), and two were reserved for laboratory baseline assessments. Of the latter, one tube was processed immediately for same-day complete blood count (CBC), erythrocyte sedimentation rate (ESR), and C-reactive protein (CRP) quantification. The second tube was centrifuged within 2 h of collection to isolate serum, which was then stored at −20 °C for subsequent enzyme-linked immunosorbent assay (ELISA) determination of target inflammatory cytokines (IL-1β, IL-8, IL-18, and TNF-α).

**Figure 1:**
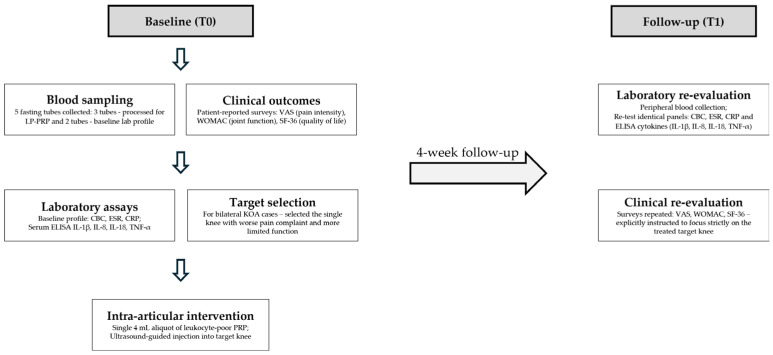
Schematic flowchart of the study design, illustrating the timeline for clinical assessments, biological sampling, and the intra-articular intervention.

The same fasting blood collection and processing protocol was repeated at the 4-week follow-up visit (T1). To minimize inter-assay variability, longitudinal samples obtained from the same participant were analyzed on the same microplate. Extracellular protein concentrations were quantified using commercially available sandwich enzyme-linked immunosorbent assay (ELISA) kits (Elabscience Bionovation Inc., Houston, TX, USA) according to the manufacturer’s instructions: high-sensitivity human IL-1β (Cat. No. E-HSEL-H0001), high-sensitivity human IL-8 (Cat. No. E-HSEL-H0004), human IL-18 (Cat. No. E-EL-H0253), and human TNF-α (Cat. No. E-EL-H0109). Absorbance was measured using a BioTek Synergy H1 Hybrid microplate reader. Cytokine concentrations were calculated from standard curves generated using a four-parameter logistic (4PL) regression model in GraphPad Prism 6.01 software. The distribution of biological samples, laboratory assays, and corresponding assessment time points is summarized in Figure 1.

The study was conducted in accordance with the principles of the Declaration of Helsinki and received approval from the University Ethics Committee (103/14 April 2025).

### 2.3. Platelet-Rich Plasma (PRP) Preparation and Injection Technique

A total of 30 mL of peripheral venous blood was obtained from each participant under aseptic conditions and distributed into three sterile 10 mL DPG PRP tubes preloaded with sodium citrate anticoagulant and a separation gel (Dermoaroma Italy S.r.l., Rome, Italy). Blood samples were processed immediately after collection.

The samples were centrifuged at 1700× *g* (approximately 4000 rpm) for 5 min to separate the blood components and facilitate platelet enrichment within the plasma fraction. Following centrifugation, the upper 1.5 mL of plasma from each tube, corresponding to the platelet-poor plasma (PPP) fraction, was carefully aspirated and discarded to increase the relative platelet concentration. Subsequently, each tube was gently tilted to an angle of approximately 90–100° ten times to detach platelets adhering to the separation gel surface and facilitate their resuspension within the remaining plasma fraction. This processing step yielded approximately 7.5 mL of platelet-enriched plasma from the three tubes combined.

To verify and characterize the cellular composition of the PRP product used in this study, a representative subset of 10 PRP preparations was selected for hematological analysis. Following homogenization and resuspension of platelets adhering to the separation gel, a 0.5 mL aliquot was collected from each preparation and analyzed using an automated hematology analyzer. These measurements were performed exclusively for PRP characterization and quality control purposes and were not part of the clinical outcome assessment. No erythrocytes were detected in any sample, while leukocyte counts ranged from 0 to 60 cells/μL, indicating minimal leukocyte contamination. Platelet counts ranged from 1,340,000 to 1,900,000 platelets/μL (1340–1900 × 10^9^/L). Baseline peripheral blood platelet counts in the corresponding participants ranged from 184 to 384 × 10^9^/L, confirming substantial platelet enrichment in the final PRP preparation (Table 1).

From this total volume, a 4 mL aliquot of the lowest, platelet-rich fraction (immediately adjacent to the separation gel interface) was carefully harvested for immediate clinical use. The remaining volume (approximately 3.5 mL) was stored for growth factor analysis within a separate parallel research project; therefore, these data are beyond the scope of the present study.

A 4 mL aliquot of platelet-rich plasma (PRP) harvested from the platelet-enriched fraction was administered intra-articularly into the target knee joint. All injections were performed by the same experienced physician via a lateral approach under strict aseptic conditions using real-time ultrasound guidance (Logiq E9, General Electric) equipped with a 6–15 MHz linear transducer and a sterile 21-gauge needle. No local anesthetic was administered. Following the injection, the knee underwent five passive flexion–extension cycles to promote uniform intra-articular distribution of the injected fluid.

Patients were advised to maintain relative rest and avoid high-impact or strenuous physical activities for 48 h after the procedure, while refraining from the use of nonsteroidal anti-inflammatory drugs (NSAIDs) for two weeks.

Patients were monitored for 30 min post-procedure for any immediate adverse reactions before being discharged with instructions regarding their scheduled 4-week (T1) clinical and laboratory follow-up evaluation.

### 2.4. Patient Evaluation

Clinical and functional assessments were performed at baseline (T0) and at the 4-week follow-up (T1), evaluating the following outcomes:

- Pain Intensity: Measured using a 100 mm Visual Analog Scale (VAS).
- Functional Status: Joint mobility, walking capacity, and the impact of the disease on activities of daily living (ADL) were evaluated using the Western Ontario and McMaster Universities Osteoarthritis Index (WOMAC) questionnaire.
- Quality of Life: Assessed using the Short Form-36 (SF-36) health survey.

To ensure data consistency, patients were actively instructed during evaluation to complete all patient-reported outcome measures (VAS, WOMAC, and SF-36) with strict reference to the injected target knee.

### 2.5. Statistical Analysis

Categorical variables are expressed as frequencies and percentages. Continuous data were evaluated for normality using the Shapiro–Wilk test; normally distributed variables are presented as mean ± standard deviation (SD), whereas non-normally distributed variables are expressed as median and interquartile range (IQR; 25th–75th percentiles).

Absolute changes (Δ) were calculated as the difference between post-treatment and baseline values (T1–T0). Relative changes were calculated as a percentage of the baseline values. Baseline (T0), post-treatment (T1), and Δ (Δ) values were visualized using box-and-whisker plots exclusively for parameters demonstrating statistically significant differences over time. Longitudinal comparisons were performed using the paired *t*-test for normally distributed data and the Wilcoxon signed-rank test for non-normally distributed data.

Bivariate associations between quantitative or ordinal variables were evaluated using the Pearson correlation coefficient in the absence of outliers, and the Spearman rank correlation coefficient otherwise. To identify independent predictors of clinical outcomes while accounting for potential confounding and preventing issues with multicollinearity, multivariable linear regression analysis was performed. For each clinical outcome variable (VAS, WOMAC, and SF-36), two distinct covariate-adjusted models were constructed, both adjusting for age, body mass index (BMI), and C-reactive protein (CRP) levels:

- **Model 1 (Baseline Profile):** Evaluated baseline levels of IL-1β, IL-8, IL-18, and TNF-α as independent predictors of the respective clinical outcomes.
- **Model 2 (Dynamic Profile):** Evaluated the absolute changes (ΔIL-1β, ΔIL-8, ΔIL-18, and ΔTNF-α) as independent variables to assess their relationship with clinical variations.

No formal correction for multiple testing was applied. The analyses consisted of Pearson correlation tests examining prespecified associations between distinct biological and clinical variables (e.g., cytokine levels, age, and clinical outcome measures), each representing a separate research hypothesis rather than multiple pairwise comparisons within the same factor. Consistent with recommendations for exploratory, hypothesis-driven analyses, adjustment procedures such as the Bonferroni correction were not implemented, as they may substantially increase the risk of type II error and obscure potentially meaningful associations.

All statistical analyses were conducted using IBM SPSS Statistics for Windows, version 25.0 (IBM Corp., Armonk, NY, USA). The statistical significance threshold (α) was set at 0.05.

## 3. Results

Among the 40 included patients, 25% were male, the mean age was 60.78 years, and 7 (17.5%) had diabetes (Table 2).

**Figure 2:**
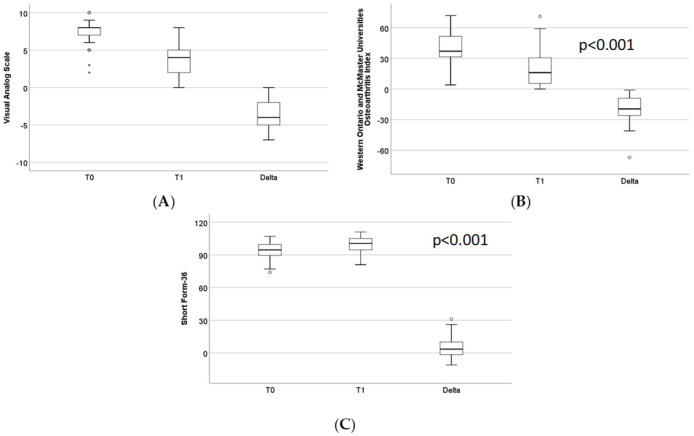
Distribution of baseline (T0), follow–up (T1), and absolute change (Δ) values for clinical outcomes and inflammatory biomarkers demonstrating statistically significant longitudinal changes. (**A**) VAS score; (**B**) WOMAC score; (**C**) SF–36 score. Boxplots represent the median, interquartile range, and minimum–maximum values.

VAS and WOMAC scores decreased significantly over time (*p* < 0.001) (Figure 2A,B). SF-36 scores increased statistically significantly over time (*p* < 0.001) (Figure 2C).

**Figure 3:**
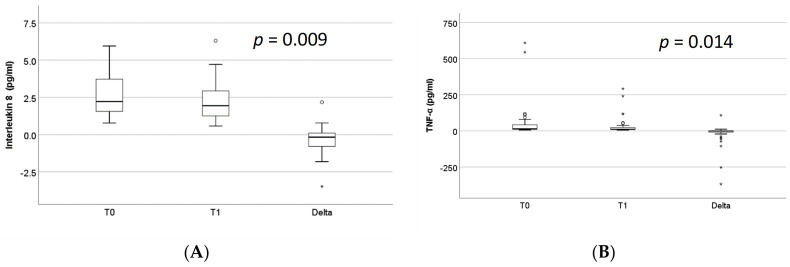
Distribution of baseline (T0), follow–up (T1), and absolute change (Δ) values for clinical outcomes and inflammatory biomarkers demonstrating statistically significant longitudinal changes. (**A**) IL–8 concentration; and (**B**) TNF–α concentration. Boxplots represent the median, interquartile range, and minimum–maximum values.

IL-1β showed a slight increase from baseline to follow-up; however, the difference did not reach statistical significance (T0: 2 (1.25; 10.9); T1: 2.2 (1.19; 10.43); *p* = 0.063). IL-8 and TNF-α decreased significantly over time, although these reductions were modest in magnitude (*p* = 0.009 and *p* = 0.014, respectively) (Figure 3A,B). IL-18 showed a small numerical decrease, which was not statistically significant (T0: 422.28 (302.97; 565.51); T1: 420.48 (327.78; 547.45); *p* = 0.667).

Table 3 presents the correlations between baseline VAS scores and WOMAC, SF-36 scores, and various demographic and clinical characteristics. A multivariable analysis was also performed with baseline VAS score as the dependent variable and baseline IL-1β, IL-8, IL-18, and TNF-α levels as independent variables, adjusted for age, BMI, and CRP. IL-1β and TNF-α were statistically significantly correlated with VAS score at baseline, but after controlling for age, BMI and CRP, the associations did not remain statistically significant.

Table 4 presents the correlations of WOMAC with SF-36 and various demographic and clinical characteristics. A multivariable analysis was also performed with baseline WOMAC score as the dependent variable and baseline IL-1β, IL-8, IL-18, and TNF-α levels as independent variables, adjusted for age, BMI, and CRP. None of them were statistically significantly correlated with WOMAC at baseline; however, after controlling for age, BMI, and CRP, that association with TNF-α became statistically significant.

Table 5 presents the correlations of SF-36 with various demographic and clinical characteristics. A multivariable analysis was also performed with baseline SF-36 score as the dependent variable and baseline IL-1β, IL-8, IL-18, and TNF-α levels as independent variables, adjusted for age, BMI, and CRP. None of them were statistically significantly correlated with SF-36 at baseline or after controlling for age, BMI and CRP.

Correlations between changes in VAS (ΔVAS), WOMAC (ΔWOMAC), and SF-36 (ΔSF-36) scores and baseline demographic or clinical variables were not reported because none reached statistical significance. After running models with ΔVAS, ΔWOMAC, and ΔSF-36 as dependent variables and IL-1β, IL-8, IL-18, and TNF-α at baseline as independent variables controlled with age, BMI, and CRP, none significantly influenced the dependent variables. We also evaluated the relationship between changes in clinical outcomes (ΔVAS, ΔWOMAC, and ΔSF-36) and changes in inflammatory biomarkers (ΔIL-1β, ΔIL-8, ΔIL-18, and ΔTNF-α) using multivariable models adjusted for age, BMI, and CRP. None of these associations reached statistical significance, except for the relationship between ΔIL-18 and ΔWOMAC (coefficient B = −0.041, 95% CI: −0.078 to −0.004, *p* = 0.032).

## 4. Discussion

The findings of this prospective observational study indicate that a single intra-articular PRP injection is associated with significant short-term improvements in pain and functional status in patients with knee osteoarthritis (KOA), occurring alongside the selective modulation of specific inflammatory markers. These early observations align with broader evidence from randomized controlled trials and meta-analyses, which have reported significant pain reduction and functional improvement after PRP treatment compared to baseline, placebo, or hyaluronic acid controls, with clinical efficacy especially beyond the six-month mark [9,23,24,25,26].

Furthermore, when evaluated against minimal clinically important difference (MCID) thresholds, the literature indicates that PRP treatment frequently exceeds established MCID thresholds for both pain relief and WOMAC functional scores at 6–12 months compared with conventional injectable therapies [23,27]. Notably, the 4-week reduction in VAS scores observed in our cohort was both statistically significant and clinically relevant, exceeding the established MCID range of 1.3–2.0 points for KOA [23,28,29,30]. This rapid initial response is consistent with previous reports in PRP-treated populations, where mean symptom reductions typically surpass clinical thresholds during early follow-up intervals [31].

On a broader scale, recent meta-analyses indicate that PRP generally provides superior pain relief compared with placebo, hyaluronic acid, or corticosteroids within the first year after treatment, though significant inter-study heterogeneity persists [23,30,32]. These differences may be related, in part, to variations in platelet concentration; higher concentrations are routinely associated with greater clinical improvement, suggesting a dose-dependent effect [33]. In parallel, comparative analyses of leukocyte-rich and leukocyte-poor formulations show that while both achieve similar improvements in pain and function, leukocyte-rich formulations trigger a higher incidence of transient local adverse reactions, supporting a more favorable safety profile for leukocyte-poor PRP [34].

The significant short-term improvement in WOMAC scores observed in our cohort aligns with the documented functional benefits of PRP, reflecting early enhancements in joint mobility and physical performance. Because functional recovery remains a primary therapeutic goal in osteoarthritis, the magnitude of these 4-week changes is comparable to that reported in recent systematic reviews [30,34]. Beyond baseline pain mitigation, the literature supports the role of PRP as a multimodal intervention capable of reducing joint stiffness and improving overall physical function [29,35]. While our study captures these clinical variations at an early post-injection endpoint, these findings are conceptually consistent with longitudinal data demonstrating sustained improvements across all WOMAC domains for up to 12 months following PRP treatment [24,35].

This multimodal therapeutic impact is well-documented in the recent literature. For instance, Chalidis et al. (2025) reported concurrent improvements in pain, in joint function, and in physical mobility, reinforcing the concept that PRP targets multiple dimensions of osteoarthritis-related disability [36]. These observations mirror earlier work by Eymard et al. (2020), which highlighted the global symptomatic benefits, including reduced stiffness and enhanced performance in daily activities [35].

In our cohort, the modest but statistically significant improvement in SF-36 scores aligns with these broader findings, corroborating the positive—albeit preliminary—short-term impact of PRP on overall health-related quality of life [24,34]. While several studies report positive trends following PRP administration, the magnitude of change in generic QoL outcomes is generally smaller and demonstrates greater variability compared to disease-specific instruments like the WOMAC or Knee Injury and Osteoarthritis Outcome Score (KOOS).

Meta-analytic data support this modest benefit, noting that post-intervention QoL changes do not consistently reach MCID thresholds, and the overall quality of evidence remains constrained [9]. Nevertheless, early randomized evidence provides valuable context; for instance, the double-blind trial by Jubert et al. (2017) demonstrated that PRP can improve overall QoL and general health domains compared to corticosteroids in patients with advanced KOA, particularly at short- to mid-term follow-up (3–6 months) [37]. These insights suggest that PRP may exert a broader patient-perceived benefit beyond localized symptom relief, even if the absolute clinical effect size remains limited at early intervals.

Recent clinical data expand on these long-term dynamics. A cohort study by Sánchez et al. (2024) indicated that repeated administrations of leukocyte-poor PRP can improve both physical and mental components of the SF-12 at 6 months, suggesting a potential cumulative or dose-related effect on perceived health status [38]. Similarly, De Matthaeis et al. (2024) reported post-treatment improvements in patient-reported QoL alongside significant reductions in pain and WOMAC scores following high-dose, neutrophil-depleted PRP, although generic QoL measures are not yet consistently implemented across standard orthobiologic protocols [39].

However, orthobiologic therapy is not universally superior to other conservative interventions. For instance, structured exercise programs frequently yield comparable or even superior improvements in certain QoL domains, highlighting the clinical importance of integrating PRP within a broader, multimodal rehabilitation framework [40]. Furthermore, high-level evidence from large, placebo-controlled trials—most notably the RESTORE trial—underscores the inherent complexity of interpreting global well-being outcomes in osteoarthritis research [41]. Even in instances where PRP does not demonstrate clear statistical superiority over placebo for specific structural and symptomatic endpoints, this landmark trial reinforces how heavily psychosocial and contextual factors influence patient-perceived global health.

Ultimately, the available literature indicates that while PRP can contribute to broader HRQoL improvements, these changes remain consistently smaller and less uniform than those observed for disease-specific outcomes such as WOMAC or KOOS. This statistical discrepancy does not necessarily denote a lack of therapeutic value; rather, it reflects the broad, multi-dimensional nature of generic instruments like the SF-36. These tools capture global health variations across multiple organ systems and psychosocial states, making them fundamentally less sensitive to acute, localized, joint-specific improvements during early follow-up intervals.

From a biological perspective, these findings were associated with selective changes in certain inflammatory biomarkers. The statistically significant reduction in circulating IL-8 levels is particularly relevant, given this cytokine’s central role in neutrophil recruitment and synovial inflammation in osteoarthritis [17]. In addition, the observed reduction in serum TNF-α levels at 4 weeks post-treatment provides valuable clinical insights. While previous studies have demonstrated reductions in inflammatory markers within synovial fluid following intra-articular PRP injections [15,42,43,44], corresponding variations within the systemic circulation remain less frequently characterized. These findings align with evidence indicating that PRP may modulate inflammatory cytokine expression and attenuate catabolic signaling within the joint environment, as well as with preclinical data suggesting a potential downregulation of systemic inflammatory markers [10,45,46,47]. Although statistically significant, the magnitude of these biomarker changes was modest, with reductions below 10% of baseline values, and their clinical relevance remains to be established. Nevertheless, the observed pattern is biologically plausible, as PRP has been reported to modulate NF-κB signaling in chondrocytes and synoviocytes, thereby attenuating downstream inflammatory pathways involving IL-1β and TNF-α [10,44].

Conversely, other inflammatory markers, including IL-1β and IL-18, did not show statistically significant changes over the 4-week follow-up period. This variability is consistent with the broader literature, which highlights the heterogeneous biological response to PRP therapy, influenced by factors such as PRP composition, disease stage, and patient-specific characteristics [48,49].

Although PRP has been shown to modulate synovial fluid cytokines, several studies evaluating peripheral blood failed to demonstrate a significant effect on systemic pro-inflammatory cytokine levels several months post-injection [16]. A primary explanation for this discrepancy is a biological floor effect: baseline plasma levels of these pro-inflammatory cytokines frequently fall near or below the assay’s limit of detection, thereby limiting the statistical detection of further reductions [16]. Additionally, systemic assessments performed weeks to months post-injection are poorly timed to capture transient, early alterations in inflammatory profiles [16,50,51]. Most fundamentally, PRP exerts predominantly local intra-articular effects within a closed compartment; given that the typical osteoarthritic joint contains only 2–4 mL of synovial fluid, any local cytokine fluctuations are unlikely to produce measurable changes once diluted within the approximately 5 L systemic circulation [10]. When systemic effects do manifest, they more frequently involve alterations in chemokines and catabolic enzymes rather than classical interleukins [50].

IL-1β and IL-18 did not change significantly during follow-up. Similar findings have been reported in previous studies evaluating systemic inflammatory markers after PRP administration [52,53]. The absence of significant changes may reflect the predominantly local action of PRP and the variability of systemic cytokine responses reported in the literature. Therefore, these findings should be considered exploratory and hypothesis-generating. The absence of uniform shifts across the entire cytokine panel suggests that the clinical benefits of PRP may not rely solely on measurable reductions in inflammatory markers [16,42,51]. Instead, therapeutic outcomes likely stem from a complex interplay of anti-inflammatory, anabolic, and regenerative pathways [10,50,51], driven by growth factor release and modulation of the synovial microenvironment [10,42,46,54]. Characterizing PRP strictly as a passive anti-inflammatory agent oversimplifies its complex biological behavior. Experimental studies have proposed that PRP may exhibit a biphasic biological response: a highly regulated initial pro-inflammatory phase required for the clearance of cellular debris, which subsequently transitions into an extended phase of inflammation resolution and tissue anabolism [10,51]. Consequently, and somewhat counterintuitively, the therapeutic success of intra-articular PRP injections may be reflected by a concomitant reduction in both pro-inflammatory (e.g., IL-6, TNF-α) and anti-inflammatory (e.g., IL-10) cytokines. This synchronous decline points to a broad immunomodulatory effect and a restoration of joint homeostasis, rather than a unilateral shift toward the overproduction of anti-inflammatory mediators [42,50].

### 4.1. Strengths and Clinical Context

An important strength of this study is its real-world observational design, which enhances external validity by reflecting routine clinical practice. These pragmatic data complement highly controlled randomized trials by providing clear insights into treatment behavior within a more heterogeneous patient population under standard clinical conditions [55].

Within the broader scope of multimodal rehabilitation frameworks for KOA—which encompass diverse physical therapy modalities, including electrical stimulation techniques targeted at pain mitigation and potential chondroprotection—intra-articular PRP injections represent a promising minimally invasive adjunct within conservative management [56,57]. By evaluating these outcomes in an everyday clinical setting, our findings contribute to the growing body of evidence documenting favorable short-term clinical trends in pain reduction and functional status, while offering preliminary observations regarding potential systemic immunomodulatory variations.

### 4.2. Limitations and Future Directions

Conversely, several key limitations of the present study must be explicitly acknowledged. First, the absence of a control or placebo arm limits our ability to definitively isolate the therapeutic effects of PRP from the substantial placebo responses characteristically associated with intra-articular interventions. Second, the potential for selection bias, the small sample size (*n* = 40), and the brief 4-week follow-up window limit the immediate generalizability and interpretation of these findings.

Furthermore, product and biological heterogeneity remain an overarching challenge in contemporary orthobiologic research. Pronounced variations in preparation protocols, final platelet yields, leukocyte subsets, and individual patient metabolic profiles complicate comparisons across the existing literature and hinder the development of standardized clinical guidelines [24,35,58,59]. Although hematological characterization confirmed substantial platelet enrichment and minimal leukocyte contamination in a representative subset of 10 PRP preparations, this analysis was performed exclusively for product characterization and quality control purposes. Therefore, potential inter-individual variability in the cellular composition of the PRP preparations administered throughout the study cannot be completely excluded. In addition, given the exploratory nature of the study and the relatively small sample size, the multivariable regression analyses should be interpreted with caution and considered primarily hypothesis-generating.

Moreover, the relatively small sample size in relation to the number of covariates included in the multivariable models may have limited the statistical power to detect modest independent associations and increased the risk of model overfitting. Consequently, the observed regression findings should be interpreted as exploratory rather than confirmatory and require validation in larger, adequately powered cohorts.

Consequently, these 4-week findings must be treated as preliminary. Future multi-center investigations utilizing larger, double-blinded randomized cohorts with extended longitudinal tracking and rigorous product characterization are strictly warranted to confirm these initial observations and validate long-term clinical efficacy.

Because the present study lacked a control or placebo group, the observed clinical improvements cannot be attributed directly to PRP treatment, and contributions from placebo effects, regression to the mean, or natural symptom fluctuations cannot be excluded.

## 5. Conclusions

The findings of this prospective exploratory study show that a single intra-articular PRP injection is associated with significant short-term improvements in pain and functional status in patients with knee osteoarthritis. The observed reductions in VAS and WOMAC scores, together with modest improvements in SF-36 outcomes, suggest that PRP may represent a potentially useful minimally invasive therapeutic option for early symptom management in routine clinical practice.

From a biological perspective, the selective reductions in circulating IL-8 and TNF-α levels indicate possible biomarker changes following PRP administration, whereas the other evaluated cytokines remained stable over the 4-week interval. However, the biological significance of these changes remains uncertain and should be interpreted cautiously, given the exploratory nature of the study.

Furthermore, while baseline inflammatory profiles did not independently predict clinical outcomes, the specific association observed between changes in IL-18 (ΔIL-18) and functional recovery (ΔWOMAC) suggests that individual cytokine dynamics may contribute to variations in early treatment response.

Overall, these preliminary 4-week findings support the potential clinical utility of PRP as an adjunctive therapeutic option for patients with mild-to-moderate knee osteoarthritis. Nevertheless, the absence of a control group, the relatively small sample size, and the short follow-up period limit the immediate generalizability of these results. Therefore, the observed clinical improvements cannot be attributed directly to PRP treatment, and potential contributions from placebo effects, regression to the mean, or natural symptom fluctuations cannot be excluded. Large-scale, double-blinded randomized controlled trials incorporating standardized PRP characterization and extended follow-up are warranted to confirm these observations and further elucidate the biological mechanisms underlying clinical response.

## Figures and Tables

**Table 1:** Hematological characterization of the PRP preparation (*n* = 10).

Parameter	Range
Platelets in peripheral blood (×10^9^/L)	184–384
Platelets in PRP (×10^9^/L)	1340–1900
Leukocytes in PRP (cells/μL)	0–60
Erythrocytes in PRP	Not detected

Data are presented as ranges.

**Table 2:** Baseline demographic and clinical characteristics of the study population.

Baseline Parameter	Study Group (*n* = 40)
Age (years)	60.78 ± 10.94
Male, *n* (%)	10 (25.0)
Kellgren–Lawrence grade, *n* (%)	
Grade 2	24 (60.0)
Grade 3	16 (40.0)
Diabetes, *n* (%)	7 (17.5)
BMI (kg/m^2^)	30.39 ± 4.98
Platelets (×10^9^/L)	267.32 ± 58.52
ESR (mm/h)	6 (5; 10)
CRP (mg/dL)	0.2 (0.1; 0.4)

Data are presented as mean ± standard deviation, median (interquartile range), or *n* (%), as appropriate. BMI, body mass index; ESR, erythrocyte sedimentation rate; CRP, C-reactive protein.

**Table 3:** Correlations and multivariable analysis of baseline VAS scores.

Variable	r	*p*	Adjusted B (95% CI)	*p*
WOMAC T0	0.35	0.028	—	—
SF-36 T0	−0.13	0.424	—	—
IL-1β T0	−0.36	0.022	−0.063 (−0.141; 0.016)	0.113
IL-8 T0	0.28	0.084	−0.050 (−0.419; 0.320)	0.786
IL-18 T0	0.26	0.101	0.002 (−0.001; 0.005)	0.156
TNF-α T0	−0.34	0.029	−0.002 (−0.007; 0.003)	0.457
Age	0.37	0.018	0.048 (−0.001; 0.097)	0.053
BMI	0.34	0.033	0.118 (0.002; 0.234)	0.047
Platelets	0.13	0.435	—	—
ESR	0.09	0.577	—	—
CRP	0.12	0.472	−1.334 (−3.297; 0.629)	0.176

r, correlation coefficient. Adjusted B values are from multivariable linear regression models adjusted for age, body mass index (BMI), and C-reactive protein (CRP). Em dashes indicate that no regression coefficient is reported for that variable in the model. ESR, erythrocyte sedimentation rate; CI, confidence interval.

**Table 4:** Correlations and multivariable analysis of baseline WOMAC scores.

Variable	r	*p*	Adjusted B (95% CI)	*p*
VAS T0	0.35	0.028	—	—
SF-36 T0	−0.36	0.024	—	—
IL-1β T0	−0.18	0.28	0.178 (−0.626; 0.982)	0.113
IL-8 T0	0.21	0.203	0.591 (−3.203; 4.384)	0.786
IL-18 T0	0.11	0.506	0.026 (−0.007; 0.058)	0.156
TNF-α T0	−0.22	0.17	−0.058 (−0.109; −0.007)	0.027
Age	0.4	0.01	0.766 (0.266; 1.267)	0.004
BMI	0.27	0.095	0.257 (−0.937; 1.451)	0.664
Platelets	0.17	0.303	—	—
ESR	0.31	0.059	—	—
CRP	0.39	0.013	13.049 (−7.097; 33.194)	0.196

r, correlation coefficient. Adjusted B values are from multivariable linear regression models adjusted for age, body mass index (BMI), and C-reactive protein (CRP). Em dashes indicate that no regression coefficient is reported for that variable in the model. ESR, erythrocyte sedimentation rate; CI, confidence interval.

**Table 5:** Correlations and multivariable analysis of baseline SF-36 scores.

Variable	R	*p*	Adjusted B (95% CI)	*p*
VAS T0	−0.13	0.424	—	—
WOMAC T0	−0.36	0.024	—	—
IL-1β T0	0.13	0.423	0.000 (−0.492; 0.392)	0.818
IL-8 T0	−0.25	0.12	0.000 (−2.592; 1.577)	0.623
IL-18 T0	0.07	0.653	0.000 (−0.019; 0.017)	0.894
TNF-α T0	0.05	0.757	0.011 (−0.017; 0.039)	0.421
Age	−0.39	0.013	−0.252 (−0.528; 0.023)	0.071
BMI	0.02	0.897	−0.041 (−0.697; 0.615)	0.899
Platelets	−0.28	0.086	—	—
ESR	−0.15	0.35	—	—
CRP	0.16	0.316	5.127 (−5.942; 16.196)	0.352

r, correlation coefficient. Adjusted B values are from multivariable linear regression models adjusted for age, body mass index (BMI), and C-reactive protein (CRP). Em dashes indicate that no regression coefficient is reported for that variable in the model. ESR, erythrocyte sedimentation rate; CI, confidence interval.

## Data Availability

The data presented in this study are available from the corresponding authors upon reasonable request. The data are not publicly available due to privacy and ethical concerns.

## Abbreviations

The following abbreviations are used in this manuscript:

ADL	activities of daily living
BMI	body mass index
CBC	complete blood count
CI	confidence interval
CRP	C-reactive protein
ELISA	enzyme-linked immunosorbent assay
ESR	erythrocyte sedimentation rate
HRQoL	health-related quality of life
IL	Interleukin
IQR	interquartile range
KOA	knee osteoarthritis
KOOS	Knee Injury and Osteoarthritis Outcome Score
MCID	minimal clinically important difference
MMPs	matrix metalloproteinases
NF-κB	nuclear factor kappa-light-chain-enhancer of activated B cells
NSAIDs	nonsteroidal anti-inflammatory drugs
PPP	platelet-poor plasma
PRP	platelet-rich plasma
QoL	quality of life
SD	standard deviation
SF-12	Short Form-12 Health Survey
SF-36	Short Form-36
TNF-α	tumor necrosis factor-alpha
T0	baseline time point
T1	follow-up time point
VAS	Visual Analog Scale
WOMAC	Western Ontario and McMaster Universities Osteoarthritis Index
4PL	four-parameter logistic (regression model)
